# Supplementary material for: Optimized braces for the treatment of adolescent idiopathic scoliosis: A study protocol of a prospective randomised controlled trial
Source: PLoS One. 2024 Feb 7;19(2):e0292069. doi: 10.1371/journal.pone.0292069 (PMC10849249; doi:10.1371/journal.pone.0292069)
Supplement: S1 Protocol — (PDF) [file pone.0292069.s002.pdf]

2022-3437: Validation of braces designed to optimize the correction of Providence type night braces

Document 02 – Research protocol (Translated from French)

D01 – List of clinical documents

1. Summary of the study

Title	Validation of braces designed to optimize the correction of Providence-type night braces
Methodology	Randomized, controlled, crossover and parallel design clinical trial.
Duration of the study	Patients will be followed for a period of two years
study center	CHU Sainte-Justine
Objective	Validate the new brace design method by optimizing immediate correction.
Number of patients	58 patients
Diagnosis and inclusion and exclusion criteria	Diagnosis: Adolescent idiopathic scoliosis<br><br>Inclusion criteria:<br>• Aged 10 and 16 inclusively<br>• Thoracic/lumbar/thoracolumbar scoliosis between 20 º and 40 º<br>• Risser index between 0 and 2<br>• <18 months after menarche<br>Exclusion criteria:<br>• Cardiovascular disease<br>• Neurological disorders (myopathy, neuropathy or central nervous system disorders)<br>• Spondylolisthesis with grade superior to 1.<br>• Other disease of the musculoskeletal system in the lower limbs.<br>• Pregnancy

2. Context

Importance of research

Adolescent idiopathic scoliosis (AIS) affects 280,000 young people in Canada, 66,000 in Quebec (prevalence 3-5%) [1], [2]. Left without follow-up or treatment, it can progress rapidly during growth and severely limit the quality of life of adolescents and adults: apparent deformity, severe low back pain (73% vs 28% in those without), early osteoarthritis, cardio-pulmonary and psychological problems[3–5].

There are only **2 recognized treatments** :

1- for moderate AIS (Cobb 25 to 40°; 10% cases, i.e. ≈25,000/year in Canada), the **brace** , thoraco-lumbo-sacral orthosis (TLSO) in rigid plastic, renewed every 6 months and worn throughout growth [6],
2- 2- for severe AIS >50° (1% cases, i.e. ≈2,500/year in Canada), surgery **with** instrumentation and fusion of the column is necessary, heavy treatment on the physical and psychological levels, which is considered last appeal.

<u>Does the brace work?</u>

The Scoliosis Research Society recommends wearing the TLSO 20-23 hrs/day until maturity in young people (Risser index 0 to 2), with a moderate curve (25-40°) [7]. Although used for >60 years, the effectiveness of the brace has been repeatedly questioned by the Canadian Task Force on Preventive Health Care [8] due to lack of evidence, leading to the withdrawal of school screening for AIS in Canada. We demonstrated that this decision was harmful [9], causing a significant increase in patients referred too late to benefit from the brace (current 19% vs 1% at screening) with surgery as the only option, without changing useless referrals (39% vs. 37%).

In 2013, the BrAIST study [10], a multi-center randomized controlled trial (RCT) confirmed the effectiveness of TLSOs: 28% of treated subjects progressed beyond 50° vs 52% of untreated (OR= 1.93 [1.08-3.46] in favor of the brace). The study, which became "Gold Standard", was interrupted by the NIH given the demonstrated superiority of the brace. A meta-analysis in 2019 reconfirmed the efficacy and safety of the treatment of idiopathic scoliosis by bracing [11].

For night braces, such as the Providence brace, a randomized study in 2014 demonstrated that these braces could reduce the progression of scoliosis in patients with curves below 25 degrees [12]. More recently, a literature review and meta-analysis showed that night braces had a success rate of 78.7% versus 73.2% for full-time braces such as TLSO braces [13]. The latest data in the literature therefore show that there is a clinical equivalence in the treatment with a TLSO brace and the night brace.

<u>New paradigm for brace-optimized treatment of adolescent idiopathic scoliosis</u>

The brace is useful but its effectiveness is not optimal [14,15], which is why our team proposed a research project with the Canadian Institutes of Health Research to optimize the treatment by brace of idiopathic scoliosis of the teenager

As part of a grant obtained from the Canadian Institutes of Health Research (Foundation grant no. 375116, 2016-2017), Dr. Hubert Labelle's team aimed through the proposed project to correct three deficiencies observed in the clinic during the brace treatment; 1- The number of patients needed to treat to achieve success is high due to the inability to predict which AIS will progress, 2-

Correction under brace is incomplete, 3- There is a significant problem with adherence to treatment . The general objective of Dr. Labelle's Foundation program was that within 7 years, brace treatment would be radically changed by doubling the % of success compared to the % of subjects needed to be treated, by the implantation of a platform unique and innovative treatment for specific and optimized care for each child. More specifically, two of the sub-goals were to increase treatment adherence (number of hours the brace was worn) by 50% and to increase the success rate of the brace by 20% using our innovative design technique. and Computer Aided Manufacturing (CAD/CAM) and finite element simulation (BraceSim). To date, the first sub-objective has been achieved through the development and validation of the first instrument for measuring functional, physical, emotional and social well-being under brace (MOBI questionnaire (My Orthopedic Brace Inventory)). This is used to identify the elements associated with non-adherence and was validated as part of a study at the CHU Sainte-Justine (project number MP-21-2018-1756 approved by the research ethic comittee (CER) dated 2017 -12-06). In the continuity of this project, we wish to complete a preliminary study in order to complete the second sub-objective aiming to increase the success rate of the brace by the use of CAD/CAM technique. In recent years, our team has developed innovative methods of computer-aided design and manufacturing (CAD/CAM) based on digital simulation models personalized for each patient (brace simulator), particularly in the context of programming the Canada Research Chair in Orthopedic Engineering and Prof. Aubin CRSND-Découverte snubvention (CER 2018-1825). A previous randomized clinical study [16] by our group demonstrated that the use of CAD/CAM methods to design braces is adequate and provides immediate correction that reduces the progression of scoliosis. The results were equivalent or superior to the BrAISt [10] study , used as a reference in the literature. The BrAISt study demonstrated by a randomized study that wearing a brace reduced the progression of scoliosis compared to observing progression without a brace alone. Since then, our group has developed a new design and simulation model described in more detail in section **Error! Reference source not found.**.

3. Review of relevant literature and work of our team

3.1. Evaluation of scoliotic deformities

Traditionally, the evaluation of scoliotic deformities is made by physical examination and with frontal X-rays. The usefulness of 3D reconstruction for the study of scoliosis is now widely accepted [17] . Our research team was a pioneer in this field by developing an innovative technique for taking calibrated postero-anterior (PA) and lateral radiographs allowing the 3D reconstruction of the internal geometry of patients with an error of 3.3± 3.8mm [18] .

This technology has been adopted and integrated to the EOS low-dose radiology system[19], an innovative technology developed in France (BioSpace, Inc) in collaboration with our team 20 . The EOS system eliminates the positioning and calibration system, and we have demonstrated that it produces x-rays 8 to 10 times less irradiating than conventional digital x-ray systems 21 . The evaluation of scoliotic deformities is made using computer tools allowing the calculation of different measurable 3D indices with low variability 22 . Our user-friendly software (Clindexia) 23 automatically measures 2D and 3D geometric indices of the spine, pelvis and trunk.

The complete characterization of scoliotic deformities also requires the study of the external geometry of the patient's trunk. This is essential since improvement in the aesthetic appearance of the trunk is the most important criterion for which patients with AIS desire improvement and consent to treatment 24,25 .

3.2. Development and validation of a computer tool for the design and manufacture of braces

The different modes of mechanical action of braces come down to the application of moments or forces on the trunk 26 since the spine is not directly accessible. How these forces are transferred to the spine is poorly documented. A few studies have measured the forces generated by the Milwaukee brace using mechanical sensors 27,28 , and those generated by the Boston brace using pneumatic and electronic sensors 29,30 . However, these studies do not present the localization of the forces at the orthosis-skin interface, nor the variation of these forces according to the position of the patients, aspects necessary for understanding the biomechanics of the orthosis.

Our team has developed a system made up of force cells connected to an electronic acquisition unit for storing data during the measurement of the tensions in the belts 31 , and we have demonstrated that the prescribed tension is very variable according to the subjects and varies greatly depending on the position of the subjects. We have also carried out the first prospective clinical study 32 to integrate the combined effect of the various parameters of the braces, i.e. the location and size of the support sites as well as the forces present, the tension of the straps and the 3D correction. obtained. Several unfavorable points of action were noted, in particular on the abdomen, at the lumbar level and posteriorly on the thorax, but on the side opposite to the deformation of the ribs. These observations may explain why there is not always a significant correction and agree with our clinical results on the 3D evaluation of the Boston brace 33 .

It is all of this work that led us to develop a computer tool for the design and adjustment of braces, integrating 3D reconstruction by x-rays, 3D reconstruction of the surface of the trunk and the brace as well as the measurement of the pressures generated by the brace on the thorax of scoliotic patients, with the aim of helping the clinician to improve the brace treatment by allowing him to visualize on screen the superimposed geometries of the bony structures, the surface of the trunk and of the brace in real time. This computer tool called Cosmescia 34 has been validated and used to perform a prospective, randomized clinical study comparing immediate 3D correction of scoliotic deformity in a group of 48 subjects with AIS whose Boston brace was adjusted with Cosmescia versus a control group whose adjustment was done by the conventional clinical method. This randomized study has clearly demonstrated that the developed tool produces better correction compared to the conventional method and that it allows for the first time a significant improvement in the 3D correction of scoliosis. These are very encouraging and exciting results that support the continuation of our research program and the upcoming dissemination of this tool in clinical settings as well as its commercialization. However, the tool remains complex to use and must be simplified for clinical use.

Over the past two decades, new methods of manufacturing braces have been developed using computer-aided design and manufacturing (CAD/CAM) techniques. The manufacturing principles are similar to conventional methods, but are computerized. The outer surface of the patient's trunk is scanned by scanner and sent to software which the orthotist can use to draw/modify the shape of the brace. A 'positive' of the shape is then generated by a computer-aided milling machine and the brace is thermo-formed on this positive. CAD/CAM is faster and produces braces that are as effective as the conventional method [36], making it a very promising biomedical field that remains underdeveloped in Canada.

Lagarrigue (www.lagarrigue.com), one of the 2 largest orthotics-prosthetics companies in France, has created such CAD software, Rodin4D, and it has partnered with Boston Brace, one of the largest manufacturers orthoses in the USA, to manufacture braces distributed in Europe and America. It seemed only natural to our team to join forces with these companies to continue our research and develop a new platform for the generation of orthoses, within the framework of an NSERC-Strategic Program grant (STPGP 380992 -09).

This manufacturing technique is now frequently used throughout the world [37].

3.3. Development of a brace simulator

Faced with the complexity of the anatomical structures of the trunk, the development of biomechanical modeling by finite elements has made it possible to study the interactions between the spine and the thorax [38–41], to analyze scoliotic deformations [42] and allowed us to simulate their orthotic treatment [41,43]. A detailed biomechanical model of the trunk, personalized to the geometry of each patient, was developed by our team, including the vertebrae, the pelvis, the rib cage, the costovertebral joints, several ligaments as well as the main muscles [40]. The adequacy between the 3D geometry predicted by the model and the geometry of the patient in his orthosis made it possible to justify the use of the model to simulate the action of the braces and to study the mechanisms of correction and the contribution of the different forces applied to the trunk [40,44–46]. Based on these studies, a treatement approach was proposed and was simulated, demonstrating that it is possible to obtain a better 3D correction by modifying the location of the support points. An optimization approach was developed and integrated into the model in order to find the location, orientation and magnitude of the forces on the trunk allowing an optimal correction. We continued these analyzes by developing a detailed explicit modeling of the brace and its interface with the trunk [47–49], in order to simulate the installation of a brace on a patient [50,51]. This model makes it possible to evaluate the effectiveness of a brace even before its manufacture. We can therefore identify any shortcomings in the design of a brace and correct them iteratively in order to optimize its effectiveness. The model makes it possible to predict what will be the geometric corrections of the scoliotic deformities inside the brace. The amplitude and location of the pressures exerted by the brace on the patient's trunk can be calculated in order in particular to detect any areas of discomfort. The model also analyzes the effect of the brace on the pathological pressures exerted on the vertebral growth plates, making it possible to estimate its capacity to halt the progression of scoliosis in the long term [52]. This model was used to evaluate the design parameters of a brace having the most influence on its effectiveness, thanks to an experimental plan where more than 12,000 braces were tested. The importance of the position of the trochanteric extension and of the opening (posterior or anterior) was particularly underlined. Finally, the link between the immediate

correction generated by the brace and its long-term effect, ie its ability to prevent the progression of scoliotic deformities, has been demonstrated from a biomechanical point of view. A strong correlation between the immediate correction of the Cobb angle of the frontal curves and the correction of the asymmetry of the pressures on the growth plates has been found [51] .

3.4. CAD/CAM of braces:

Thanks to an agreement with the industrial partners Lagarrigue in France, Boston Brace in the USA and Orthèses-Prothèses Rive-Sud in Montreal, developed within the framework of the NSERC Strategic Program grant, we have coupled our 3D trunk reconstruction system (EOS and surface scanner) and our brace simulator to the CAD/CAM software (RODIN 4D) from Lagarrigue, so that we have a platform allowing the design/simulation/optimization and computer-aided manufacturing of TLSO orthoses. This CAD/CAM platform provides a synthesis of the various advanced technologies developed by the industry and by our research group in order to improve the effectiveness of brace treatment [16] .

4. Objective and assumptions

The general objective of the project is to validate the new CAD/CAM brace design method integrating the optimization of immediate correction.

Primary Hypothesis (H1) : After 1 year and 2 years of use, the out-of-brace correction, as measured by Cobb's angle, from the Test braces will be non-inferior to the Control braces. The non-inferiority margin will be 5 degrees.

Secondary hypothesis (H2) : The immediate and medium-term (1 and 2 years) failure rate of treatment will be 10% lower for Test braces compared to Control braces. There are three different types of failures:

1. Mid-term failure (1 and 2 years) – relative progression : A progression of the out-of-brace Cobb angle of 5 degrees or greater than the value measured at visit 1.
2. Intermediate (study duration) failure – absolute progression : A progression of the Cobb angle of the main off-brace curvature to greater than 45 degrees (SRS failure criterion).
3. Medium-term failure (duration of the study) – surgery : Stopping treatment with a brace and a decision to have surgery (AVBGT or fusion).

Secondary hypothesis (H3) : The quality of life of patients wearing Test braces in the medium term, as measured with the SRS-22r questionnaire, will be non-inferior to that of patients wearing control braces. The non-inferiority margin will be 10%.

Secondary hypothesis (H4) : The immediate in-brace correction of the Cobb angle of the Test brace will be no less than that of the control brace. The non-inferiority margin will be 5 degrees.

The effects measured (dependent variables) are as follows:

Effects measures	Descriptions
Major	
Cobb angle off brace (Initial, 6 months, 1 year, 2 years).	Measure using low-dose EOS radiography<br>to assess the evolution of scoliosis.
Secondary	
Cobb angle<br>in the brace. (Initial)	Measure using digital radiography to assess the immediate corrective effect of braces.
Treatment failure rate (Initial, 6 months, 1 year, 2 years)	See failure criteria in section 6.5
The lordosis and kyphosis effect of the brace (Initial, 1 year, 2 years)	The angles of the curves in the sagittal plane will be measured on the x-ray images.
The number of hours wearing the brace per day (Initial, 1 year, 2 years)	A compliance monitor will measure the rate of brace use.
The patient's quality of life (Initial, 6 months, 1 year, 2 years)	The quality of life of patients will be calculated from the SRS-22r questionnaire

5. Descriptions of braces

This section describes the two different types of brace, control brace and test brace, which will be used.

5.1. Control Brace

The control brace will be made using the method currently used at Sainte-Justine Hospital. It will therefore be designed by an experienced orthotist using scanning of the patient's skin's outer surface, and Rodin4D, a 3D design platform. The brace will be adapted to include a compliance monitor. The compliance monitor is an iButton thermal sensor (*Boston Brace, MA, USA*), this will record the wearing time of the brace. This will in no way affect the correcting effect of the

brace. It will then be manufactured by thermoforming in the facilities of Orthèse-Prothèse Rive-Sud.

5.2. Test Brace

The test brace results from a combination of semi-automatic generation process and iteration of simulations of the immediate effect of the brace in order to guide the modification of the shape and to improve the topology of the brace before manufacturing.

The test brace will be designed following a new design method. First, a personalized finite element model (FEM) will be created from 3D reconstructions of the patients' spine, pelvis, rib cage and sternum, on which the geometry of the skin is registered and connected by deformable elements simulating the patient's soft tissues. From this basic configuration, the total straightening of the spine in the frontal plane and the alignment of the vertebrae in the transverse plane will be simulated, resulting in a deformed trunk geometry. The surface of the trunk will then be extracted and cut using anatomical landmarks of the skeleton to give the preliminary shape of the brace.

The tightening of the initial brace will then be simulated in the FEM: the geometry of the brace is superimposed on that of the patient and a gradual tightening of the straps is imposed. Contact elements between the surface of the trunk and the inside of the brace transmit the tightening forces of the brace to the patient's model until a position of equilibrium is reached. By extracting the compressive forces acting on the vertebral epiphyseal growth plates out of the brace and into the tight brace the immediate brace correction is simulated.

In order to systematize the design and converge towards an optimal design, the initial brace is modified in an iterative process, using the results of the immediate simulation. Modifications can be made manually (by manually modifying the brace topology) or automatically (by following an optimization process). At each iteration, the brace is simulated by the FEM and its immediate effectiveness is evaluated by an objective function combining the simulated 3D corrections/evolution (immediate and post-growth) and the pressures exerted on the skin.

The optimization automatically iterates different brace designs and evaluates the objective function for each new design. By using global optimization algorithms (substitute optimization, shape recognition, etc.), these changes are guided to converge towards an optimal solution that would maximize the effectiveness of the brace according to the objective function. Several simplifications must be implemented in order to ensure that a solution is reached within a reasonable time. Therefore, at the final stage of the design process, the optimal brace found will be checked and refined manually to ensure its sufficient resolution, smoothness and feasibility of manufacture. The iteration process will be performed by a graduate student in engineering under the supervision of an orthopedic surgeon.

Proof of concept and preliminary results

The process for generating optimized braces was tested numerically on eight cases by comparing their correction efficiency with that of reference braces. The design time required was five minutes for the semi-automatic process and approximately 1h30 for the reference brace. For these preliminary results, the immediate lumbar correction was significantly better (55% vs 31%, p = 0.004) for an equivalent level of pressure exerted on the trunk.

The immediate correction simulation method has already been carried out by our group [53] . It had been shown that the position lying on the back made it possible in part to correct the deformation of the column and that the Providence-type brace came to rebalance the forces on the growth plates of the vertebrae.

The brace will be adapted to include a compliance monitor. This will in no way affect the correcting effect of the brace. The compliance monitor is an iButton thermal sensor (*Boston Brace, MA, USA*), this will record the wearing time of the brace. The new type of brace (test brace) will be manufactured in the same way by CAM and thermoforming in the facilities of Orthèse-Prothèse Rive-Sud .

6. Methodology

6.1. Study design

A prospective, double-blind, non-inferiority, controlled and randomized clinical trial will be conducted to assess the effectiveness of a new orthopedic brace design platform. Fifty-eight patients will be recruited at the CHU Ste-Justine orthopedic clinic. The study will be divided into two phases. The first phase will use a crossover design to assess the immediate correction of both types of brace. The second phase will use a parallel design with a 1:1 allocation to assess the mid-term correction.

In phase 1, each patient will try on each type of brace with a “control: test” or “test: control” trial sequence. Immediate correction in braces will be assessed. During phase 2 each patient will use one of the two types of braces for a period of two years. There will be a renewal of the brace after 6 or 12 months based on updated spine x-rays and a new trunk geometry. This is in order to consider the growth of the patient and the inherent changes in the patient's body and the scoliotic deformity. Taking into account that all patients do not follow the same growth, renewal of the brace may sometimes be necessary before 12 months (for example after 6 months following a rapid growth spurt). In this case, the brace will be renewed before the end of 12 months to ensure adequate follow-up of the treatment by the patient. If the patient is part of the test group, the optimization of the correction will be redone.

6.2. Recruitment method

All potential subjects presenting to the scoliosis clinics of the CHU Sainte-Justine will be recruited on a voluntary basis, through the clinical research unit in orthopedics (URCO). Potential candidates will be met by the URCO nurse coordinator who will provide them with the study information and consent form, answer questions from the patient and/or parents, and direct them to the orthopedist after agreed to join the study and signed the consent form. The characteristics and motivations of the subjects refusing to take part in the study will be kept in order to verify the presence of a selection bias in the sample if this number turns out to be significant. According to our previous experience [35], the vast majority of subjects agree to participate in this type of study.

6.3. Inclusion and exclusion criteria

Inclusion criteria: Patients (girls or boys) must have received a confirmed diagnosis of adolescent idiopathic scoliosis (thus aged between 10 and 16 years inclusive) regardless of the type of curve (I to VI) according to the Lenke classification [54]. All subjects will be candidates for brace treatment according to the criteria recommended by the Scoliosis Research Society [7] : thoracic/lumbar/thoracolumbar scoliosis between 20 ° and 40 ° , as measured by the Cobb technique on a PA radiograph; a Risser index between 0 and II (before skeletal maturity); <18 months after menarche .

Exclusion criteria: Will be excluded from the study, subjects with a history of cardiovascular disease, neurological disorders, including myopathy, neuropathy or disorders of the central nervous system, or any other disease of the musculoskeletal system at the level of the legs. Patients with spondylolisthesis of grade greater than 1 will be excluded from the study. Patients with pregnancy will also be excluded from the study.

The use of these standard criteria for the indication of brace treatment in this randomized controlled clinical trial ensures that we have generalizable results and constitutes an ethical approach since this study concerns the majority of the population of patients eligible for brace treatment.

6.4. Method and follow-up visits

Visit 1: Acquisition of parameters

During the initial visit, the following interventions will be performed :

- An EOS low-dose digital biplanar radiograph (EOS-Imaging, Paris, France) will be acquired: simultaneous AP and lateral while the patient is standing in a comfortable position, with hips and knees extended, both elbows bent with the hands at the side of the head. A student will digitize the anatomical landmarks on each x-ray, so as to obtain a 3D reconstruction of the spine, pelvis and rib cage. A scanning technician, blind to the purposes of our study and the subject's allocation group, will verify and approve each of the reconstructions. The relevant geometric variables will be obtained automatically on

Clindexia, eliminating any possibility of radiological measurement bias by a human observer.

- Curve flexibility will be qualitatively assessed by the orthopedist to determine if the patient has a flexible, normal, or rigid curve
- A scan of the external surface of the trunk will be acquired with the TechMed3D surface scanner.
- The SRS-22r questionnaire will be administered. This questionnaire is administered to patients in the scoliosis clinics at the CHU Sainte-Justine even if they are not part of a research project at all visits (except for the delivery of a brace). The questionnaire is administered through the AMIKO platform (platform developed by the CHU Sainte-Justine to computerize the questionnaires at the orthopedic clinic), or the questionnaire is administered in paper version.

Design and manufacture of braces

Before the second visit, which will take place approximately one month after the first, each patient will have a control brace and a test brace designed and manufactured according to the specific geometry of their trunk and spine as described in Section 5.

Visit 2: Cross-testing of the braces and delivery of the brace in the medium term

During Visit 2, each patient will sequentially try on both braces. This corresponds to phase #1 (crossover design) of the study mentioned in section 6.1. The following interventions will be made:

The first brace will be delivered, and the location of the corrective supports, the overall conformation of the brace and the tension of the straps will be checked by the orthotist for the control brace and for the test brace. Neither the orthopedist nor the orthotist can modify the corrective effect of the test brace in their adjustments. The same manipulations will then be repeated with the second brace in the randomization sequence.

A digital x-ray will be taken in a supine position with the patient in a comfortable position, with the hips and knees extended, the hands in a lateral position relative to the head. The Cobb angle will be measured by an evaluator blind to the aims of the study. The same manipulations will then be repeated with the second brace in the randomization sequence.

- The patient will receive the brace corresponding to the group assigned to him by the randomization for phase #2. He will also receive the usual instructions for wearing the brace, unless he meets a failure criterion (see section 6.5).

Follow-up visit

Follow-up visits will take place approximately every six months (more or less 2 months). They will make it possible to follow the progression of the treatment and the evolution of the quality of life. The following actions will be performed at each follow-up visit:

- A pair of low-dose EOS digital x-rays will be acquired: simultaneous AP and lateral while the patient is standing in a comfortable position, with hips and knees extended, hands lateral to the head.
- The SRS-22r questionnaire will be administered.

If necessary, a renewal of brace can be prescribed, according to the judgment of the orthopedist and the orthotist. If the brace needs to be renewed, the following additional interventions will be performed:

- A scan of the external surface of the trunk will be acquired with the TechMed3D surface scanner.

Brace renewals

If a brace renewal is prescribed, the new brace will be designed according to the initial design method (control or test) detailed in section 5. The brace will be returned to the clinic approximately one month later, following the usual procedure. During the brace delivery, the following actions will be performed:

- Minor adjustments to cutouts and topology to ensure patient comfort following the same procedure as original delivery
- Installation of an iButton to monitor wearing time
- A digital x-ray will be acquired in a supine position with the patient in a comfortable position, with hips and knees extended, hands in a lateral position relative to the head.

6.5. Failure criteria

The failure criteria for the treatments are those mentioned in secondary hypothesis H2.

Note that a patient in phase #1 is likely to have immediate failure of the brace assigned to them for phase #2. In this case, for ethical reasons, the brace of the other group will be assigned to him. Also for ethical reasons, a patient may have to use the brace of the other group during phase #2 if it offers an immediate correction of the Cobb angle greater than 5 degrees.

7. Randomization

Patients who meet all the inclusion criteria and no exclusion criteria will be randomized twice independently.

Randomization #1 will be to determine the trial sequence of brace types at Visit #2. Randomization #1 allocation will have a 1:1 ratio for the “test:control” sequence and the “control” sequence. : test ".

Randomization #2 will be to determine the brace type the patient will use for a period of two years after the visit. The allocation will have a ratio of 1:1 for test and control brace type respectively.

The randomization will be done by the URCO nurse coordinator according to a block allocation of 2, 4 or 6. Neither the engineers nor the orthopedist nor the orthotist will know the brace assigned for phase #2 until these are designed, manufactured and adjusted.

8. Statistical analysis plan

8.1. Sample size justification

The sample size was calculated to be able to accept the main hypothesis (H1) with a probability of 5% of committing the type I error and a power of 80%. The calculation was performed with nQuery software (GraphPad Software DBA Statistical Solution, San Diego, CA) and the following parameters were used:

- Significance level$\alpha = 5\%$
- Allocation ratio$\kappa = 1$
- Margin of non-inferiority$\delta = 5°$
- standard deviation$\sigma = 7°$ [16]
- Power$(1 - \beta) = 80\%$

The sample size should be 25 per group, so a total of 50 patients. On the other hand, a loss to follow-up of 15% is anticipated. We will therefore need 29 patients per group for a total of 58 patients.

8.2. Types of analyzes

There will be two types of data analysis: an intention-to-treat (ITT) analysis and a per-protocol (PP) analysis. Data analysis by ITT will include all patients who have been randomized. A study on patient compliance shows that, on average, patients wear the night brace 90.7% of the prescribed time (generally 8 hours) [55] . For this reason there will also be an analysis of the results by PP which will only include patients who have worn the brace on average for more than 7.2 hours per day. This method of analysis will make it possible to evaluate the correction of the brace if it is worn with good compliance.

8.3. Analysis of primary hypothesis H1

The analysis of primary hypothesis H1 will have the off-brace Cobb angle as the dependent variable and the type of brace worn as the independent variable. The 95% confidence interval of the difference in correction between the test brace and the control brace will be constructed and compared to the non-inferiority margin.

8.4. Analysis of secondary hypothesis H2

The analysis of secondary hypothesis H2 will have the treatment failure rate as the dependent variable and the type of brace worn as the independent variable. The incidence of failures will be presented with a Kaplan-Meier curve, the statistical analysis will be done with a log-rank test.

8.5. Analysis of secondary hypothesis H3

The analysis of secondary hypothesis H3 will have the respective scores of the SRS-22r questionnaire as the dependent variable and the type of brace worn as the independent variable. The 95% confidence interval of the difference in score between the test brace and the control brace will be constructed and compared to the non-inferiority margin.

8.6. Analysis of secondary hypothesis H4

A statistical analysis will initially be completed to verify that there is no residual effect in the crossover design of the study. The analysis of secondary hypothesis H4 will have the Cobb angle while wearing the brace as the dependent variable and the type of brace as the independent variable. The 95% confidence interval of the difference in score between the test brace and the control brace will be constructed and compared to the non-inferiority margin.

9. Ethical considerations

The research protocol will be submitted to the CHU Sainte-Justine Research Ethics Committee (CER). The participation of patients in the project will be free and voluntary. The research team undertakes to respect the confidentiality of the data and the anonymity of the participants. To do this, the data will be coded and stored securely at URCO. More information on data storage can be found in the document: Y:\URCO\Etude Clinique Brace Providence Optimise\01-Documents\D01-Liste_Documents_Cliniques.

Patients who refuse to participate in the study will be offered conventional treatment for their scoliosis to ensure that all patients receive appropriate treatment for their condition.

9.1. Risks

Patients participating in this study will receive one more digital X-ray than patients receiving conventional treatment. The risk remains low since the other x-rays are based on the EOS dose. They have up to 8-10 times less radiation than a standard X-ray [21] . The additional X-ray seems to us to be justified given the low radiation associated with the other X-rays, because it makes it possible to compare the immediate effectiveness of the two types of braces and to ensure that a patient is not randomized in an inferior treatment.

Participants will be dressed in light, tight-fitting clothing, which could be inconvenient for some.

There is also the risk that the optimized brace will be less effective or less comfortable. The effectiveness of the optimized brace is unknown.

9.2. Benefits

Participants may benefit from improved treatment with the optimized brace if it proves superior to the control brace.

The participation of participants in the clinical study will increase knowledge about braces. This knowledge will be published and orthotists can use it to improve their brace treatment.

10. References

1. Canada S. Statistics Canada.
2. Konieczny MR, Senyurt H, Krauspe R. Epidemiology of adolescent idiopathic scoliosis. *J Child Orthop* . 2013;7(1):3-9.
3. Payne III WK, Ogilvie JW, Resnick MD, Kane RL, Transfeldt EE, Blum RW. Does scoliosis have a psychological impact and does gender make a difference? *Spine (Phila Pa 1976)* . 1997;22(12):1380-1384.
4. Weinstein SL, Zavala DC, Ponseti I V. Idiopathic scoliosis. Long-term follow-up and prognosis in untreated patients. *J Bone Jt Surg-Ser* A. 1981;63(5). doi:10.2106/00004623-198163050-00003
5. Mayo NE, Goldberg MS, Poitras B, Scott S, Hanley J. The Ste-Justine Adolescent Idiopathic Scoliosis Cohort Study. Part III: Back pain. *Spine (Phila Pa 1976)* . 1994;19(14):1573-1581.
6. Labelle H. Orthotic treatment of pediatric spinal disorders and diseases. *Spine State Art* Rev. 1990;4:1-13.
7. Richards BS, Bernstein RM, D'Amato CR, Thompson GH. Standardization of criteria for adolescent idiopathic scoliosis brace studies: SRS Committee on Bracing and Nonoperative Management. *Spine (Phila Pa 1976)* . 2005;30(18). doi:10.1097/01.brs.0000178819.90239.d0
8. Care CTF on PH. New grades for recommendations from the Canadian Task force on Preventive Health Care. *CMAJ* . 2003;169(3):207-208.
9. Beauséjour M, Roy-Beaudry M, Goulet L, Labelle H. Patient characteristics at the initial visit to a scoliosis clinic: A cross-sectional study in a community without school screening. *Spine (Phila Pa 1976)* . 2007;32(12). doi:10.1097/BRS.0b013e318059b5f7
10. Weinstein SL, Dolan LA, Wright JG, Dobbs MB. Effects of Bracing in Adolescents with Idiopathic Scoliosis. *N Engl J Med* . 2013;369(16). doi:10.1056/nejmoa1307337
11. Zhang Y, Li X. Treatment of bracing for adolescent idiopathic scoliosis patients: a meta-analysis. *Eur Spine* J. 2019;28(9):2012-2019. doi:10.1007/s00586-019-06075-1
12. Wiemann JM, Shah SA, Price CT. Nighttime bracing versus observation for early

adolescent idiopathic scoliosis. *J Pediatric Orthop* . 2014;34(6):603-606. doi:10.1097/BPO.0000000000000221
13. Costa L, Schlosser TPC, Jimale H, Homans JF, Kruyt MC, Castelein RM. The Effectiveness of Different Concepts of Bracing in Adolescent Idiopathic Scoliosis (AIS): A Systematic Review and Meta-Analysis. *J Clin Med* . 2021;10(10):2145. doi:10.3390/jcm10102145
14. Donzelli S, Zaina F, Minnella S, Lusini M, Negrini S. Consistent and regular daily wearing improve bracing results: a case-control study. *Spinal Scoliosis Disorder* . 2018;13(1):1-8.
15. Hawary R El, Zaaroor-Regev D, Floman Y, Lonner BS, Alkhalife YI, Betz RR. Brace treatment in adolescent idiopathic scoliosis: risk factors for failure—a literature review. *Spine* J. 2019;19(12). doi:10.1016/j.spinee.2019.07.008
16. Guy A, Labelle H, Barchi S, et al. Braces Designed Using CAD/CAM Combined or Not With Finite Element Modeling Lead to Effective Treatment and Quality of Life After 2 Years: A Randomized Controlled Trial. *Spine (Phila Pa 1976)* . 2021;46(1). https://journals.lww.com/spinejournal/Fulltext/2021/01010/Braces_Designed_Using_CAD_CAM_Combined_or_Not_With.3.aspx
17. Emans JB. Boston brace. *SRS Bracing Man* . Published online 2003.
18. Delorme S, Petit Y, De Guise JA, Labelle H, Aubin CÉ, Dansereau J. Assessment of the 3-D reconstruction and high-resolution geometrical modeling of the human skeletal trunk from 2-D radiographic images. *IEEE Trans Biomed Eng* . 2003;50(8). doi:10.1109/TBME.2003.814525
19. Dubousset J, Charpak G, Dorion I, et al. [A new 2D and 3D imaging approach to musculoskeletal physiology and pathology with low-dose radiation and the standing position: the EOS system]. *Bull Acad Natl Med* . 2005;189(2):287-300.
20. Després P, Beaudoin G, Gravel P, De Guise JA. Physical characteristics of a low-dose gas microstrip detector for orthopedic x-ray imaging. *Med Phys* . 2005;32(4). doi:10.1118/1.1876592
21. Deschênes S, Charron G, Beaudoin G, et al. Diagnostic imaging of spinal deformities: Reducing patients radiation dose with a new slot-scanning X-ray imager. *Spine (Phila Pa 1976)* . 2010;35(9). doi:10.1097/BRS.0b013e3181bdcaa4
22. Labelle H, Dansereau J, Bellefleur C, Jéquier JC. Variability of geometric measurements from three-dimensional reconstructions of scoliotic spines and rib cages. *Eur Spine* J. 1995;4(2):88-94.
23. Labelle H, Aubin CE, Jackson R, Lenke L, Newton P, Parent S. Seeing the spine in 3D: How will it change what we do? *J Pediatric Orthop* . 2011;31(1 SUPPL.). doi:10.1097/BPO.0b013e3181fd8801
24. Goldberg CJ, Gillic I, Connaughton O, et al. Respiratory function and cosmesis at maturity in infantile-onset scoliosis. *Spine (Phila Pa 1976)* . 2003;28(20). doi:10.1097/01.BRS.0000085367.24266.CA
25. Pratt RK, Burwell RG, Cole AA, Webb JK. Patient and parental perception of adolescent idiopathic scoliosis before and after surgery in comparison with surface and radiographic measurements. *Spine (Phila Pa 1976)* . 2002;27(14). doi:10.1097/00007632-200207150-00012

26. Panjabi MM, White III AA. Basic biomechanics of the spine. *Neurosurgery* . 1980;7(1):76-93.
27. Wong MS, Mak AFT, Luk KDK, Evans JH, Brown B. Effectiveness and biomechanics of spinal orthoses in the treatment of adolescent idiopathic scoliosis (AIS). *Prosthet Orthot Int* . 2000;24(2). doi:10.1080/03093640008726538
28. Mulcahy T, Galante J, DeWald R, Schultz A, Hunter JC. A follow up study of forces acting on the Milwaukee brace on patients undergoing treatment for idiopathic scoliosis. *CLINORTHOP* . 1973;93. doi:10.1097/00003086-197306000-00008
29. Chase AP, Bader DL, Houghton GR. The biomechanical effectiveness of the boston brace in the management of adolescent idiopathic scoliosis. *Spine (Phila Pa 1976)* . 1989;14(6). doi:10.1097/00007632-198906000-00018
30. Lou E, Raso JV, Hill DL, Durdle NG, Mahood JK, Moreau MJ. The daily force pattern of spinal orthoses in subjects with adolescent idiopathic scoliosis. *Prosthet Orthot Int* . 2002;26(1). doi:10.1080/03093640208726622
31. Aubin CE, Labelle H, Ruszkowski A, et al. Variability of strap tension in brace treatment for adolescent idiopathic scoliosis. *Spine (Phila Pa 1976)* . 1999;24(4). doi:10.1097/00007632-199902150-00010
32. Mac-Thiong JM, Petit Y, Aubin CÉ, Delorme S, Dansereau J, Labelle H. Biomechanical Evaluation of the Boston Brace System for the Treatment of Adolescent Idiopathic Scoliosis: Relationship between Strap Tension and Brace Interface Forces. *Spine (Phila Pa 1976)* . 2004;29(1). doi:10.1097/01.BRS.0000103943.25412.E9
33. Labelle H, Dansereau J, Bellefleur C, Poitras B. Three-dimensional effect of the Boston brace on the thoracic spine and rib cage. *Spine (Phila Pa 1976)* . 1996;21(1). doi:10.1097/00007632-199601010-00013
34. Bellefleur C, Aubin CE, Cheriet F, Labelle H. Validation of a clinical tool for brace design and adjustment. In: *7th IRSSD Meeting, Vancouver* . Flight 10.; 2004:12.
35. Labelle H, Bellefleur C, Joncas J, Aubin CÉ, Cheriet F. Preliminary evaluation of a computer-assisted tool for the design and adjustment of braces in idiopathic scoliosis: A prospective and randomized study. *Spine (Phila Pa 1976)* . 2007;32(8). doi:10.1097/01.brs.0000259811.58372.87
36. Wong MS, Cheng JCY, Lo KH. A comparison of treatment effectiveness between the CAD/CAM method and the manual method for managing adolescent idiopathic scoliosis. *Prosthet Orthot Int* . 2005;29(1). doi:10.1080/17461550500069547
37. Wong M. Measurement, Fabrication and Fitting Principles. *The Atlas* . Published online 2017:139.
38. Sundaram SH, Feng CC. Finite element analysis of the human thorax. *J Biomech* . 1977;10(8). doi:10.1016/0021-9290(77)90104-X
39. Andriacchi T, Schultz A, Belytschko T, Galante J. A model for studies of mechanical interactions between the human spine and rib cage. *J Biomech* . 1974;7(6). doi:10.1016/0021-9290(74)90084-0
40. Aubin CE, Descrimes JL, Dansereau J, Skalli W, Lavaste F, Labelle H. GEOMETRIC MODELING OF THE RACHIS AND THORAX FOR THE BIOMECHANICAL ANALYSIS BY FINITE ELEMENT OF SCOLIOTIC DEFORMATIONS. *Ann Chir* . 1995;49(8).

41. Gignac D, Aubin CE, Dansereau J, Poulin F, Labelle H. Biomechanical study of new orthotic treatment concepts for 3D correction of scoliosis. *Ann Chir* . 1998;52(8).
42. Stokes IAF, Bigalow LC, Moreland MS. Measurement of axial rotation of vertebrae in scoliosis. *Spine (Phila Pa 1976)* . 1986;11(3). doi:10.1097/00007632-198604000-00006
43. Gignac D, Aubin CÉ, Dansereau J, Labelle H. Optimization method for 3D bracing correction of scoliosis using a finite element model. *Eur Spine* J. 2000;9(3). doi:10.1007/s005860000135
44. Aubin CE, Dansereau J, De Guise JA, Labelle H. Study of the biomechanical coupling between the vertebral column and the rib cage in the treatment of scoliosis by orthosis. *Ann Chir* . 1996;50(8).
45. Aubin CÉ, Dansereau J, De Guise JA, Labelle H. Rib cage-spree coupling patterns involved in brace treatment of adolescent idiopathic scoliosis. *Spine (Phila Pa 1976)* . 1997;22(6). doi:10.1097/00007632-199703150-00010
46. Perié D, Aubin CE, Petit Y, Beauséjour M, Dansereau J, Labelle H. Boston brace correction in idiopathic scoliosis: A biomechanical study. *Spine (Phila Pa 1976)* . 2003;28(15). doi:10.1097/00007632-200308010-00008
47. Clin J, Aubin CÉ, Lalonde N, Parent S, Labelle H. A new method to include the gravitational forces in a finite element model of the scoliotic spine. *Med Biol Eng Comput* . 2011;49(8). doi:10.1007/s11517-011-0793-4
48. Clin J, Aubin CÉ, Parent S, Labelle H. Biomechanical modeling of brace treatment of scoliosis: Effects of gravitational loads. *Med Biol Eng Comput* . 2011;49(7). doi:10.1007/s11517-011-0737-z
49. Périé D, Aubin CE, Lacroix M, Lafon Y, Labelle H. Biomechanical modeling of orthotic treatment of the scoliotic spine including a detailed representation of the brace-torso interface. *Med Biol Eng Comput* . 2004;42(3). doi:10.1007/BF02344709
50. Clin J, Aubin CÉ, Parent S, Ronsky J, Labelle H. Biomechanical modeling of brace design. In: *Studies in Health Technology and Informatics* . Flight 123.; 2006.
51. Clin J, Aubin CÉ, Sangole A, Labelle H, Parent S. Correlation between immediate in-brace correction and biomechanical effectiveness of brace treatment in adolescent idiopathic scoliosis. *Spine (Phila Pa 1976)* . 2010;35(18). doi:10.1097/BRS.0b013e3181cb46f6
52. Clin J, Aubin CE, Labelle H, Parent S. Immediate correction required to expect a long-term effectiveness of a brace treatment: a biomechanical insight. *Scoliosis* . 2010;5(S1). doi:10.1186/1748-7161-5-s1-o63
53. Sattout A, Clin J, Cobetto N, Labelle H, Aubin CE. Biomechanical assessment of providence nighttime brace for the treatment of adolescent idiopathic scoliosis. *Spine Deform* . 2016;4(4):253-260.
54. Lenke LG. The Lenke Classification System of Operative Adolescent Idiopathic Scoliosis. *Neurosurg Clin N Am* . 2007;18(2). doi:10.1016/j.nec.2007.02.006
55. Antoine L, Nathan D, Laure M, Briac C, Jean-François M, Corinne B. Compliance with night-time overcorrection bracing in adolescent idiopathic scoliosis: Result from a cohort follow-up. *Med Eng Phys* . 2020;77:137-141. doi:10.1016/j.medengphy.2020.01.003
