# Supplementary material for: Optimized braces for the treatment of adolescent idiopathic scoliosis: A study protocol of a prospective randomised controlled trial
Source: PLoS One. 2024 Feb 7;19(2):e0292069. doi: 10.1371/journal.pone.0292069 (PMC10849249; doi:10.1371/journal.pone.0292069)
Supplement: S1 Document — (PDF) [file pone.0292069.s003.pdf]

Machine Translated by Google

APPROUVÉ PAR LE COMITÉ D'ÉTHIQUE
09 FÉV. 2023
#2022-3437
CHU SAINTE-JUSTINE

[redacted]

RESEARCH INFORMATION AND CONSENT FORM

Title: Validation of orthopedic corsets designed to optimize scoliosis correction adolescent idiopathic.

Responsible researcher: Hubert Labelle, MD1

Collaborating people:

- Carl-Éric Aubin, Ph. D.1,2 -
Christian Bellefleur, M.Sc.A, Research Associate1,2 -
Maxence Coulombe, B. Eng., Master's student1 - Josée
Dubois, MD, Radiologist1 - Sylvain
Deschênes , PhD, Medical Physicist1 - Julie
Joncas, Inf. B.Sc.1 - Stefan
Parent, MD, Ph.D.1 - Olivier
Chémaly, MD1 - Félix
Brassard, MD1 - Nikita
Cobetto, Ph.D. Research associate2 - Soraya
Barchi, B.Sc Research associate1 - Isabelle
Turgeon , B.Sc.Research associate1 - Marjolaine
Roy-Beaudry, M.Sc. Research associate1 - Aymeric Guy, M.
Eng, Ph. D student1, 2

- 1 CHU Sainte-Justine, 3175 Côte Sainte-Catherine Road, Montreal, H3T 1C5 2
- Polytechnic School of Montreal, 2900 Édouard-Montpetit Boulevard, Montreal, H3T 1J4

Machine Translated by Google

Source of funding :

This project is funded by the Canadian Institutes of Health Research (CIHR)

WHY ARE YOU INVITED TO PARTICIPATE IN THIS RESEARCH PROJECT?

Today, we are asking for your child's participation in this research project, carried out at the CHU Sainte-Justine, in collaboration with the École Polytechnique de Montréal, the goal of which is to improve treatment for children with idiopathic scoliosis.

Before accepting that your child participate in this research project, we invite you to read this information form in order to decide if you are interested in participating in this research project.
It is important to fully understand this form. Do not hesitate to ask questions. Take all the time you need to decide.

We encourage parents to include their child in the discussion and decision-making to the extent that the child can understand.

Your doctor is one of the investigators in this research project. As such, he is above all concerned with your well-being and also with the accomplishment of this research project. Before participating or at any time during the project you may wish to obtain the opinion of a physician who is not participating in this study. You are under no obligation to participate in any study offered to you.

WHY ARE WE CONDUCTING THIS RESEARCH PROJECT?

Your child has idiopathic scoliosis. Some scoliosis requires treatment. In the majority of cases, these scoliosis are treated by wearing a corset. Our team has developed computer software that aims to improve the design, fitting, optimization and manufacture of corsets by attempting to better personalize the corsets to each patient by simulating the spine.

In this project, we want to validate this new method of computer-assisted treatment.

HOW MANY PEOPLE WILL PARTICIPATE IN THE RESEARCH PROJECT?

Approximately 58 patients will be recruited for the entire project. All patients will be recruited at the CHU Sainte-Justine.

HOW WILL THE RESEARCH PROJECT TAKE PLACE?

During your visit to the orthopedic clinic your child will take an EOS X-ray and then see their doctor, this is part of the standard follow-up for scoliosis. You are being offered to be part of this research project because your doctor has determined that an orthopedic brace would be the appropriate treatment for your child.

Machine Translated by Google

If you agree to participate, the **first step** will be to determine the flexibility of your child's spine. To do this, the orthopedic surgeon will make a visual qualitative assessment.

The **second step** will then be for your child to answer a questionnaire (SRS-22r) to measure how scoliosis affects their quality of life.

The **third step** is your child's trunk surface scan. This step allows to obtain a 3D image to make the design of the corset digitally. This step is done in the standard corset fabrication and corresponds to the last step of the first visit.

Between the first and second visit, randomly (neither the family nor the doctor can choose which group your child will be in) your child will be assigned to use a corset designed either by the standard method or by the test method The standard corset is designed using current design techniques. The test corset is designed by a technique that digitally simulates the spine. The chances of being assigned to a group are 1 in 2. Two corsets (one by the standard method and another by the simulation method) will be made.

The **fourth step** will be done during the second visit. Your child will receive two corsets (one standard and one test) which will be adjusted. At 30-minute intervals, a radiographic acquisition (standard x-ray) will be made for each corset. This will allow the immediate effectiveness of the corsets to be assessed and will serve to verify that the corsets are effective enough to be maintained throughout your child's treatment. Your child will therefore receive an additional low-dose X-ray by participating in the study. In the **fifth stage,** your child will go

home with only one corset, the one that has been assigned to him, thus becoming his definitive corset for long-term treatment. We install a thermal sensor in the corset that measures the time your child wears the corset. Your child will be instructed to wear their corset at night, approximately 8 hours a day.

You and your child will not be informed of the type of corset assigned. Corsets are randomly assigned to participants such that one out of two participants receives an optimized corset and the other receives a standard corset. In the unlikely event that the orthotist and the attending orthopedist are not satisfied with the effectiveness obtained by the optimized and/or standard brace which would have an immediate correction lower than that envisaged, your child will de facto receive the brace from the other group and will remain in the study. If the two corsets are not satisfactory according to the orthotist and the orthopaedist, your child will receive an alternative treatment and will not be included in the study

There will be **follow-up visits** to assess the mid-term effectiveness of the brace. These visits take place at intervals between 6 months and 1 year depending on the growth of your child. A corset renewal could take place if necessary. These visits are routine in the scoliosis clinic and are already part of the standard treatment plan at our hospital. For the research project, your child will be asked to complete a quality of life questionnaire (SRS-22r) at each follow-up visit.

Machine Translated by Google

Measurements as well as the adjustment of the corsets will be carried out during standard visits for the follow-up of your child. The total time required for the search should not exceed 2 hours and 40 minutes.

In case of discomfort from the brace or for any other problem related to your child's scoliosis, you can make an appointment at the clinic before the scheduled follow-up date.

The research project will be carried out at the CHU Sainte-Justine.

The research team will consult your medical file to obtain the information relevant to this research.

HOW LONG WILL PARTICIPATION IN THIS RESEARCH PROJECT LAST?

Your child will be followed for a period of 24 months or less. Your child's brace belongs to him. He can keep it, without wearing it, once the treatment is finished.

WHAT ARE THE RISKS?

Participants will be dressed in light, tight-fitting clothing, which could be inconvenient for some. There is also the risk that the optimized brace will be less effective or less comfortable. The effectiveness of the optimized corset is unknown.

The protocol also provides for the acquisition of a standard X-ray in addition to those provided for in the standard follow-up protocol. This x-ray will be done while wearing the brace. This increases exposure to ionizing radiation. On the other hand, for the out-of-brace x-rays, we opted for an imaging system designed to limit radiation doses at the source.
Indeed, the latest generation EOS™ imaging system is said to have a “very low radiation dose”.
It differs from conventional radiographic imaging techniques by a very marked reduction in the dose of X-rays delivered to the patient.

A study measuring the radiation dose has shown that the dose received at the level of the skin is reduced by a factor of 8 to 10 with the EOS™ system compared to conventional radiology for frontal and lateral spine examinations. This corresponds approximately to the equivalent of a week's exposure to the dose of natural radiation in Montreal. Therefore, the dose of radiation to which your child will be exposed will not exceed the dose deemed acceptable. It is not associated with cardiogenic effects. As a safety measure, however, we will ensure that female participants are not pregnant during the examination by asking them orally.

ARE THERE ADVANTAGES OF PARTICIPATING IN THIS RESEARCH PROJECT?

It is impossible to predict whether your child will receive health benefits from participating in this research. However, his orthopedist may use the information collected to plan the conduct of his treatment or improve the effectiveness of his brace. In addition, the participation of your

Machine Translated by Google

child will allow us to improve the knowledge and possibly the treatment of patients with adolescent idiopathic scoliosis.

WHAT ARE THE COSTS?

The treatment will be paid for by the Régie de l'Assurance Maladie du Québec (RAMQ). The additional corset will be financed by Orthèse Protèse Rive-Sud.

In the event of harm resulting from the treatments and procedures required by this research, your child will receive all necessary medical care covered by the Régie d'assurance- maladie du Québec or by its drug insurance plan. You will have to pay the portion of the costs that are not covered.

HOW IS PRIVACY ENSURED?

During your participation in this research project, the physician in charge of the project and the research team will collect, in a research file, the information concerning you and necessary to meet the scientific objectives of the research project.

This information may include information in your medical records [including your gender and date of birth], your past and present medical condition, and the results of any tests, examinations and procedures that will be performed.

All data collected will remain confidential within the limits provided by law. You will only be identified by a code number. The key to the code linking your name to your research file will be kept by the doctor in charge of this research project.

To ensure your safety, a document attesting to your participation is placed in your medical file. In addition, the results of certain tests carried out for research purposes could be placed there depending on the context. Therefore, any person or company to whom you give access to your medical file will have access to this information.

The research data will be kept for at least 10 years after the end of the study by the doctor in charge of this research project.

Research data may be published or discussed scientifically, but will not identify you. For the purposes of monitoring, control,

protection, safety and authorization of the drug under study by the regulatory bodies, your research file as well as your medical file may be consulted by a person authorized by the regulatory bodies, in Canada or abroad, such as Health Canada, as well as by authorized representatives of the sponsor, the institution or the research ethics board. These people and organizations will have access to your personal data, but they adhere to a privacy policy.

Machine Translated by Google

You have the right to consult your research file to verify the information collected and have it corrected if necessary. Furthermore, access to certain information before the end of the research project could imply that you are withdrawn from the project in order to preserve its integrity.

ARE YOU FREE TO PARTICIPATE?

Your child's participation in this research project is free and voluntary. Any new knowledge likely to call into question the decision that your child continues to participate in the research will be communicated to you.

You can remove your child from this search at any time. Should the case be, the person in charge of the study, the information and the radiological images already collected within the framework of this research project will nevertheless be kept, analyzed or used to ensure the integrity of the research project and your safety. No new data will be collected.

Whatever your decision, it will not affect the quality of the health services offered to him.

Any new knowledge acquired during the course of the research project that could have an effect on your decision to continue to participate in it will be communicated to you quickly.

CONTACTS

For more information about this research, contact Soraya Barchi, research associates at CHU Sainte-Justine, at 514-345-4931 ext. 4352.

For any information on your child's rights as a participant in this research project, you can contact the Local Service Quality and Complaints Commissioner of the CHU Sainte Justine at (514) 345-4749.

WHERE CAN I GET MORE INFORMATION?

Clinical Trials: A description of this clinical trial will be available at http://www.clinicalTrials.gov, as required by US and Canadian law. This site will not contain any information that can identify you. At most, the site will present a summary of the results. You can search at any time.

You will be able to request a summary of the results of the research project; these will not be available until the project is fully completed.

You will receive a signed copy of this form. You can ask questions to the research team at any time.

Machine Translated by Google

CONSENT AND CONSENT

Title of the research project: Validation of orthopedic corsets designed to optimize the correction of adolescent idiopathic scoliosis.

They explained to me the nature and progress of the research project. I have read the consent form and I have been given a copy. I had the opportunity to ask questions which were answered. After reflection, I agree to participate or that my child participates in this research project.

I authorize the research team to consult my medical file, or my child's medical file, to obtain information relevant to this project.

By signing this consent form, you do not waive any of your legal rights.
In addition, you do not release the investigators and the promoter from their legal and professional liability in the event of a situation that would cause you harm.

______________________	______________________	__________
Child's name<br>(block letters)	Assent of the child capable of<br>understand the nature of the project<br>(signature<br>Or<br>Verbal assent obtained by:<br>______________________	Date

______________________	______________________	__________
Name of parent, guardian<br>(block letters)	Consent (signature)	Date

Machine Translated by Google

If applicable

___________________________ ___________________________ ___________

Name of participant aged 18 and over Consent (signature) Date

(block letters)

I explained to the participant and/or his/her parent/guardian all relevant aspects of the research and answered the questions they asked me. I told them that participation in the research project is free and voluntary and that participation can be stopped at any time.

___________________________ ___________________________ ___________

Name of person obtaining consent (signature)
Date

(block letters)

Machine Translated by Google

Addendum to Consent Form

Participant who has reached the age of 18

Title of the research project: Validation of orthopedic corsets designed to optimize the correction of adolescent idiopathic scoliosis.

I have seen today the consent that my parents had signed at the time of my entry into this research project and a copy of this signed consent was also given to me today.

I agree to continue my participation in this research project.

I understand that my participation is free and voluntary and that I can withdraw from this research project at any time.

I authorize the research team to consult my medical file to obtain information relevant to this project.

In the event of withdrawal, the data already collected will nevertheless be kept, analyzed or used to ensure the integrity of the research project and your safety. No new data will be collected.

________________	________________	__________
Participant's name	Signature	Date
________________	________________	__________
Name of the person who obtains consent	Signature	Date
